# Ultralow-Dose Adjunctive Methadone with Slow Titration, Considering Long Half-Life, for Outpatients with Cancer-Related Pain

**DOI:** 10.1089/pmr.2020.0034

**Published:** 2020-07-10

**Authors:** Srini Chary, Amane Abdul-Razzak, Lyle Galloway

**Affiliations:** Division of Palliative Medicine, Department of Oncology, University of Calgary, Calgary, Alberta, Canada.

**Keywords:** analgesia, cancer pain, methadone, pain, palliative care

## Abstract

***Background:*** The unique properties of methadone make it attractive for use in cancer pain. The use of very low initial doses of adjunctive methadone is a promising strategy given its simplicity and potentially reduced risk profile.

***Objective:*** To understand if an ultralow-dose (ULD) methadone protocol (1 mg by mouth daily initial dose with gradual titration) can improve pain control in outpatients with cancer-related pain not responsive to previous opioids and/or nonopioid analgesics. We also sought to assess if the use of ULD methadone resulted in improvement in mood and sleep among other outcomes.

***Design and Setting/Subjects:*** This study is a retrospective chart review of outpatients at the cancer pain clinic at the Tom Baker Cancer Centre in Calgary, Alberta, Canada.

***Measurements:*** The mean ratings in maximum and average pain before methadone initiation, and at the final follow-up point are reported. Paired sample *t* tests evaluate for statistically significant differences in pain ratings before methadone initiation and at final follow-up. We also report the proportion of participants with a subjective improvement in pain, sleep, and mood (dichotomous “yes/no”), and the mean number of weeks to initial documented pain improvement.

***Results:*** 68.6% of patients (24/34) reported a subjective improvement in pain. Most patients reported improved sleep and mood (78.8% and 64.7%, respectively).

***Conclusions:*** More than two-thirds of patients reported an improvement in pain with a protocol using very low initial doses of adjunctive methadone. Our report is a preliminary retrospective chart review and larger prospective trials are warranted.

## Background

Methadone is a synthetic opioid that was first produced in the 1930s^1^ and is available as a racemic mixture comprising two enantiomers, R and S, each exhibiting unique properties. R-methadone has mu and delta agonist opioid properties, whereas *S*-methadone is an *N*-methyl-d-aspartate (NMDA) antagonist and a serotonin and norepinephrine reuptake inhibitor as nonopioid properties. The racemic mixture has a long and highly variable half-life of 7–150 hours.^2^ The unique properties of methadone, namely NMDA antagonism and serotonergic and norepinephrine activity, make it a promising drug for use in cancer pain.^2–5^

Present evidence-supported methods for use of methadone involve complete rotations off previous opioids and onto relatively high doses of methadone as the sole opioid.^6–9^ Although these methods are used in inpatient palliative care settings, they may be associated with unstable pain control in the transition period, sedation, and narcosis, and usually require inpatient monitoring.^10^ The use of very low initial doses of adjunctive methadone is a promising strategy given its simplicity, potentially reduced risk profile and the addition of a drug with unique properties that are not offered by other opioids.

A 2019 systematic review found only seven studies on the use of low-dose methadone, with variable dosing strategies.^11^ Our study looks at a relatively unique protocol that starts at “ultralow doses” (ULDs) of 1 mg once daily with a slowly titrated regimen thereafter. The objective is to understand if the use of a ULD methadone protocol can improve pain control in outpatients with cancer-related pain not responsive to previous opioid and/or nonopioid analgesic treatments. We also sought to assess if the use of ULD methadone resulted in improvement in mood and sleep and allowed for a reduction in the primary opioid or other adjuvant analgesics.

## Methods

### Study design and setting

This study is a retrospective chart review of outpatients at the cancer pain clinic at the Tom Baker Cancer Centre in Calgary, Alberta, Canada. The study period was from January 2013 to May 2019.

### Study participants

Adults aged ≥18 years seen at the Tom Baker Cancer Centre Pain Clinic with pain caused by cancer or cancer treatments and who received ULD methadone and slow titration treatment were included in this study. This clinic sees patients with active cancer as well as those who have previously been treated for cancer and are in remission but continue to have ongoing chronic pain (e.g., due to cancer treatments or surgery). ULD methadone treatment involves starting at a dose of 1 mg by mouth before bedtime and is titrated weekly for the first four to five weeks and then as needed (see Appendix A1 for dose titration schedule). We excluded patients who were on methadone before being seen at the clinic, patients prescribed methadone for reasons other than analgesia, patients started on higher doses of adjunctive methadone, and those who did not fill the prescription of methadone or who did not take any doses (based on chart notes).

### Variables

Baseline characteristics were extracted from patient charts, including age, gender, type of pain (nociceptive, neuropathic, or mixed), cancer type and stage, and cancer treatments received during methadone intervention period. Other data extracted include the type of opioids used and dose, converted to morphine-equivalent daily dose (MEDD), adjuvant analgesics used and dose, and the presence, type, and severity of any reported analgesic-related adverse effects. The conversion from other opioids to MEDD utilized the following rations: 2:1 for oxycodone to morphine; and 5:1 for hydromorphone to morphine. The drug conversion ratios for fentanyl to morphine corresponded to the dose conversion tables found in fentanyl transdermal drug monograph documents (e.g., 25 mcg/h patch is equivalent to 60–134 mg oral morphine) and the mid-point of each range was utilized (e.g., 25 cmg/h patch = 97 mg oral morphine). These variables were collected both at baseline (before methadone initiation) and at the final follow-up time.

We also collected the final documented dose of methadone, pain rating at baseline and at the final follow-up point on a scale from 0 to 10 (0 = “no pain”; 10 = “worst pain”), the time to initial documentation of pain improvement on methadone, and patient reports of improvement in sleep or mood at the last follow-up point (dichotomous “yes/no” variable). More specifically, in person or phone follow-ups initially occurred weekly for the first four to five weeks and then every two to three weeks or sooner if issues arose such as poorly controlled pain or adverse effects. Patients were able to call the clinic office if questions or issues arose. The clinical practice is to ask if mood “has improved” at each follow-up time. Clinic team members also ask if sleep has improved (“yes or no”) at each of these follow-up times. Finally, at each follow-up patients are asked to rate their “worst pain” for that day and their “average” pain that day from 1 to 10, with 10 being the worst pain they have ever experienced.

### Statistical methods

Descriptive summaries are presented for baseline demographic variables. The mean ratings in maximum and average pain before methadone initiation, and at the final follow-up point are reported. Paired sample *t* tests evaluate for statistically significant differences in pain ratings before methadone initiation and at final follow-up. The mean MEDD of the primary (nonmethadone) opioid at baseline and at the time of final follow-up are presented along with Wilcoxon signed-rank test to assess for statistically significant differences in MEDD at baseline versus final follow-up. The proportion of participants on nonopioid analgesics at baseline and at final follow-up point was also calculated. We also report the proportion of participants with a subjective improvement in pain, sleep, and mood (dichotomous “yes/no”), and the mean number of weeks to initial documented pain improvement.

## Results

A total of 35 patients are included in this study with a mean age of 61 years. Twenty-one patients (61%) were female and 17 (48.6%) received cancer treatment during the study period. 74.3% of patients had advanced cancer (stage III–IV), with the most common cancer types being gastrointestinal (22.9%); gynecological (20.0%); and multiple myeloma (17.1%). More than half (54.3%) of patients had mixed pain types, and 45.7% had neuropathic pain as the sole type of pain.

68.6% of patients (24/34) reported a subjective improvement in pain, with a mean of 5.4 weeks to initial pain improvement. At baseline, three patients (9%) were not using another strong opioid. At the final follow-up point, this increased to 15 patients (42.9%). Mean follow-up duration was 55.6 weeks. The final follow-up time was defined by one of the following: (1) the last time the patient was seen in clinic before death or discharge from the clinic; (2) the last clinical follow-up within the study timeline.

Ten patients (28.6%) discontinued methadone at a mean of 19.2 weeks with reasons, including sedation, patient preference, dizziness, nausea, concerns regarding opioid diversion, intolerance, and unspecified. There were no methadone-related hospitalizations or deaths. The mean difference in maximum pain rating before versus at the final follow-up time was −2.6 (standard deviation [SD] 2.15), with paired sample *t* testing reaching statistical significance (*t* = 7.0; *p* < 0.001). Patients' rating of “average pain” before and after methadone treatment showed a mean difference of −1.4 (SD 2.1) with a statistically significant paired *t* test value (*t* = 3.9; *p* = 0.001). Most patients reported improved sleep and mood (78.8% and 64.7%, respectively) and 22.9% reported at least one adverse effect (Table 1).

**Table 1.** Outcomes of Ultralow-Dose Methadone Treatment

**Outcome**	
Subjective report of pain improvement? (*N* = 35)	*N* (%)
Yes	24 (68.6)
No	10 (28.6)
Unknown (lost to follow-up)	1 (2.9)
No. of weeks to documented initial pain improvement (*N* = 32)	*N* (%)
Mean (SD)	5.4 (3.5)
Improved sleep? (*N* = 33)	*N* (%)
Yes	26 (78.8)
No	7 (21.2)
Improved mood? (*N* = 34)	
Yes	22 (64.7)
No	12 (35.3)
Adverse effects (*N* = 35)	*N* (%)
Yes^a^	8 (22.9)
Dizziness	5
Nausea	3
Fatigue	1
Depression	1
No	20 (57.1)
Unknown/missing data	7 (20)

^a^ Some patients experienced more than one adverse effect; therefore, sum of all adverse effects is >8.

**FIG. 1.**
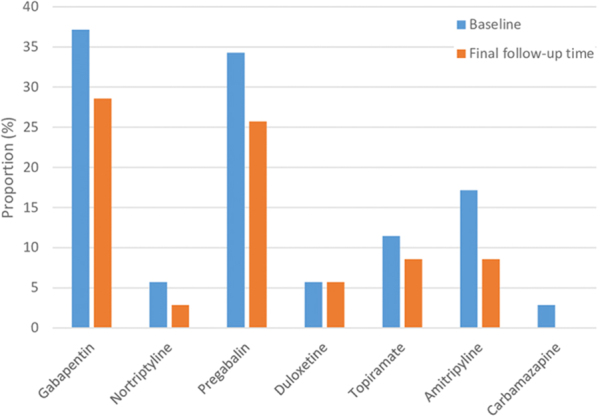
Proportion of patients on co-analgesic medications.

The median primary (nonmethadone) opioid dose, expressed as MEDD, at baseline was 65 mg (interquartile range [IQR] 40–120) and at the final follow-up point was 20 (IQR 0–34). Wilcoxon signed-rank tests showed a statistically significant difference between these two time points (*Z* = −4.7; *p* < 0.001). The median total daily adjuvant methadone dose at the point of final follow-up was 9 mg (IQR 3–23). The proportion of patients on co-analgesics was slightly reduced for most medications at the final follow-up time compared with baseline (Fig. 1).

## Discussion

In our study on the use of ULD methadone for outpatients with cancer-related pain, we found an improvement in pain in 68.6% of study participants, with statistically significant reductions in pain scores. In addition, the median dosing of other opioids was significantly reduced with the addition of adjunctive methadone and sleep and mood improved in 78.8% and 64.7% of cases, respectively. In 33.9% of patients, patients were able to discontinue their previous opioid, although the intent was not to rotate or switch completely to methadone.

The use of low-dose adjunct methadone with gradual titration reduces the need for inpatient monitoring and can reduce adverse effects such as sedation and narcosis.^12,13^ Our study is unique because an ultralow initial dose of 1 mg by mouth once daily, with gradual weekly titration considering methadone as a racemic mixture comprising two enantiomers opioid and nonopioid properties while existing opioid continued. The half-life of nonopioid methadone isomer is around 150 hours and Robert Twycross several decades ago suggested using half-life for morphine and using “on the clock” was considered, while existing opioid is continued till adjunct methadone response is noted. This strategy appears to be successful in most patients we studied, with a statistically significant reduction in maximum and average pain ratings. Our result aligns with a 2019 systematic review that found a statistically significant effect in all studies using low-dose methadone as either an adjunct or sole agent.^11^ However, we found that 28.6% of patients eventually discontinued methadone, and in most cases this was due to methadone-related adverse effects. Previous studies on adjunctive methadone are limited by loss of follow-up or otherwise have not reported discontinuation rates, making it challenging to compare our discontinuation rates with those of others.^13–15^ Another unique feature of our study is the demonstration that most patients reported improved sleep and mood, which play an important role in overall quality of life. Despite using ULD and gradual titration, existing opioid remained and methadone tissue saturation and alpha-1 globulin latching in circulation seem to have not affected the positive response of patients.

Our study included a heterogeneous group of cancer patients both on and off treatments, and with active cancer or in remission, which not only improves generalizability of results but also limits comparison with other studies focused on adjunct methadone in the palliative care setting. Just under half of the patients were receiving cancer treatment while being seen in the clinic, whereas others had completed treatment before being seen in the pain clinic. Limitations are those that may be expected of a retrospective study, including limitations in data collection and the inability to control for many important confounding variables. Finally, our sample size was small, and our report is a preliminary presentation of clinical experience. Larger prospective trials are warranted.

At this time, the evidence base for adjunct methadone in populations with palliative care needs is limited. Thus, we feel this study was important to assess if ULD methadone could be helpful and perhaps a safer strategy in the outpatient setting. It may be that a higher initial dose may be a more expedient strategy with quicker time to onset of pain relief. In their study on adjunct methadone for cancer pain, Courtemanche et al.^14^ used an initial mean starting dose of 3.5 mg and found that the median time to response was seven days, which is shorter than the time to response in our cohort, but caution must be exercised in making indirect comparisons between studies. Our study is preliminary and only a retrospective look at clinical experience. Prospective studies are needed to evaluate efficacy and safety, including studies comparing different strategies for prescribing adjunctive low-dose methadone. A strategy that balances safety and the unnecessary prolongation of suboptimal pain control needs to be identified.

Given the ability to use low-dose adjunctive methadone in a less-supervised outpatient setting, and the fact that it is an economical opioid choice, future research could focus on the use of this novel opioid in low- to middle-income countries.

Some of the challenges with methadone relate to its complex pharmacokinetics with interindividual variability. At high doses and in susceptible populations, methadone interacts with several commonly used drugs and may lead to unpredictable sedation, respiratory depression, and cardiac conduction abnormalities (QT interval on ECG [QT/QTc] prolongation, with high doses of methadone is associated with sudden death). In addition, opioid equianalgesic doses are not easy to calculate as previous studies have suggested wide variability in equianalgesic dosing.^16–19^ Our ULD method acknowledges all these uncertainties and may be more palatable for health care providers who hesitate to prescribe it at higher doses.

## Abbreviations Used

IQR: interquartile range
MEDD: morphine-equivalent daily dose
NMDA: *N*-methyl-d-aspartate
SD: standard deviation
ULD: ultralow dose

## Appendix

### Appendix A1. Methadone Dose Schedule and Titration

(Tom Baker Cancer Centre Pain Clinic—Internal Protocol)

Each subsequent step is only pursued if the previous dose is not adequate in alleviating pain and the patient is not experiencing intolerable or unsafe adverse effects.

1. Methadone 1 mg at bedtime for seven days.
2. 1 mg BID for seven days.
3. 1 mg TID for seven days.
4. 2 mg TID for seven days.
5. 2.5 mg PO TID for seven days.
6. 5 mg PO TID for seven days.

Depending upon efficacy and/or side effects further escalation or de-escalation occurred at the discretion of the prescribing physician and clinic pharmacist team. Adjustments to the timeframe of the incremental steps can also be made based on the patient's experience.

(Availability of oral methadone tablet in Canada: 1 mg, 5 mg, 10 mg, and 25 mg (hence half pill of 5 mg tablet 2.5 mg).
